# Comprehensive Optimization of Western Blotting

**DOI:** 10.3390/gels9080652

**Published:** 2023-08-14

**Authors:** Dishiwen Liu, Haoliang Wu, Shengyu Cui, Qingyan Zhao

**Affiliations:** 1 Department of Cardiology, Renmin Hospital of Wuhan University, Wuhan 430060, China; liudshw12@whu.edu.cn (D.L.); 2020203020016@whu.edu.cn (H.W.); 2018203020015@whu.edu.cn (S.C.); 2 Cardiovascular Research Institute, Wuhan University, Wuhan 430060, China; 3 Hubei Key Laboratory of Cardiology, Wuhan 430060, China

**Keywords:** immunoblotting, electrophoresis, electrotransfer, quick blocking, gel cut

## Abstract

Western blotting is one of the most extensively used techniques in the biomedical field. However, it is criticized by many researchers due to its considerable time consumption, multiple steps, and low method results. Therefore, we modified the steps of gel preparation, electrophoresis, electrotransfer, blocking, and gel cutting. First, we simplified the gel preparation step by premixing various reagents and varying the amounts of catalysts or radical generators, which shortened the entire process to 10 min. Second, we shortened the electrophoresis process to 35 min by modifying the formula of the electrophoresis running buffer. Then, we removed the hazard of methanol vapor by replacing methanol with ethanol in the electrotransfer buffer. Finally, the use of polyvinylpyrrolidone-40 shortened the blocking procedure to 10 min. Our modifications shortened the time, improved the experimental productivity, and minimized the experimental cost without hindering compatibility with most existing equipment. The entire experiment up to primary antibody incubation can be completed within 80 min.

## 1. Introduction

Western blotting (WB) is an essential tool in protein analytical chemistry [1,2]. However, its shortcomings, such as considerable time consumption, multiple steps, and poor reliability, are criticized by researchers [3,4]. Even small mistakes in any of its steps may significantly alter the entire subsequent experiment. Therefore, researchers who invested considerable time and effort in mastering this approach do not necessarily obtain satisfactory results.

The principle of immunoblotting is not particularly complicated. First, mixed antigen samples are separated by unidirectional or bidirectional electrophoresis on a gel. Then, the single antigenic component in the gel is transferred to the blotting membrane and solidified by natural adsorption, an electric field, or other external forces of the blotting membrane. Finally, the antigen-immobilized matrix membrane is detected and analyzed by using immunoisotope probes or immunoenzymatic probes [5]. However, these simple principles require various steps.

Immunoblotting includes core steps, such as gel preparation, electrophoresis, electrotransfer, blocking, and antibody incubation. Each step lasts hours or days. Despite the fact that commercial tricine-based precast gels have been developed [6,7], a high price is not appropriate for high-output experiments. Therefore, primitive methods, such as adding reagents one by one and slow solidification, are still used to formulate gels in most laboratories. In addition, traditional electrophoresis (stacking gel: 80 V; separation gel: 120 V) takes at least 90 min, and wet electrotransfer also requires 90 min. At least 60 min are required for skim milk blocking before primary antibody incubation [8]. This five-hour-long experimental process truly needs to be optimized.

Although all-in-one instruments, such as ProteinSimple Wes, are gradually gaining ground in the market [9,10,11], their high price is prohibitive for many laboratories. Other semi-automatic systems, such as SNAP i.d.* 2.0 [12] and Invitrogen™ iBind™, have been developed to accelerate the protein detection process at the expense of consuming large quantities of precious antibodies.

No matter how often these instruments are upgraded, the fundamentals of immunoblotting remain the same. In this work, we modified the gel preparation, electrophoresis, membrane transfer, blocking, and gel cutting steps. While being compatible with most existing instruments, our modifications shorten the time needed, improve the experimental success rate and productivity, and also decrease the experimental cost. The entire experiment can be completed within 80 min before the primary antibody incubation, which dramatically improves the labor and productivity of the experimenter.

## 2. Results

### 2.1. Pre-Mixed Reagents Simplify and Accelerate Gel Preparation

**Figure 1:**
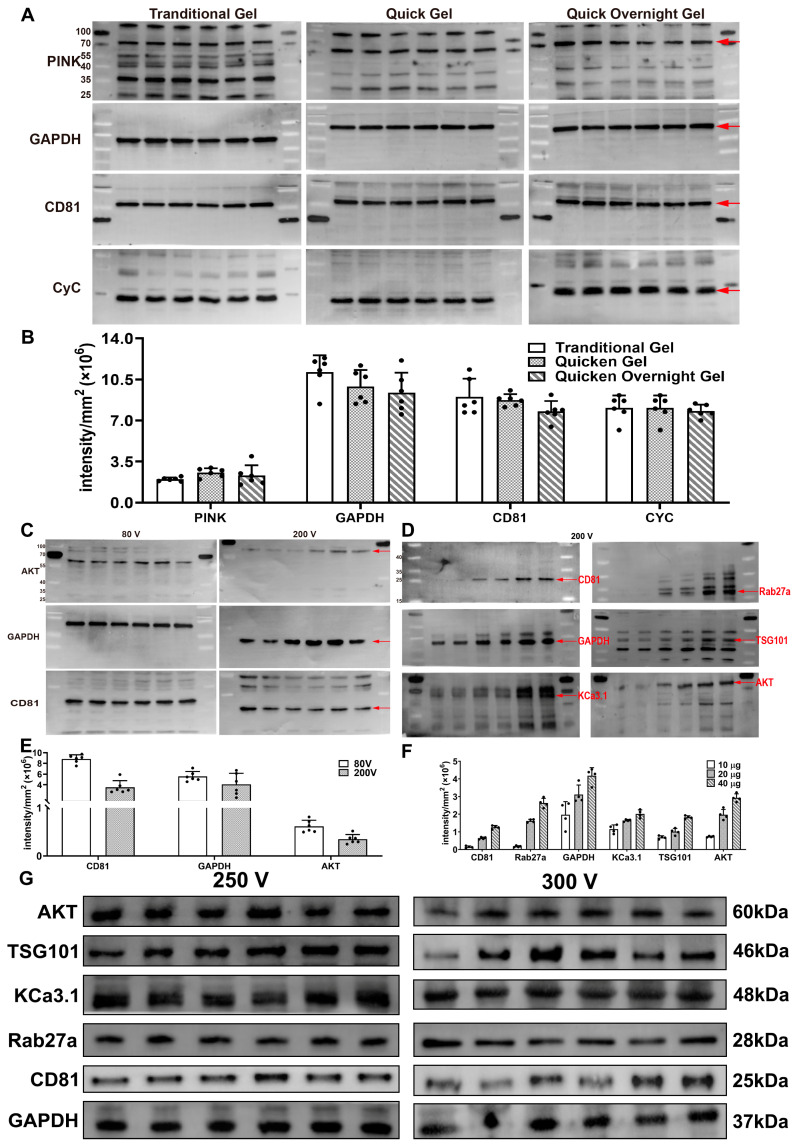
Pre-mixed gels and modified electrophoresis running buffer accelerate the experimental procedures. (**A**) Comparison of signal intensities after the development of traditional gels, quick-formulated gels, and quick-formulated gels at 4 °C overnight. The experimental procedure was the same as the standard experimental procedure except for the gels. (**B**) Signal intensities after the development of different gel preparation schemes were statistically analyzed and compared (n = 6). There was no substantial difference in signal-to-noise ratio between the gels prepared using the different methods. (**C**) The traditional electrophoresis running buffer tested at 200 V. The experimental procedure was the same as the standard experimental procedure except for the electrophoresis running buffer. (**D**) Modified electrophoresis running buffer tested at 200 V. The experimental procedure was the same as the standard experimental procedure except for the blocking reagent (Epizyme, Shanghai, China). (**E**) Signal intensities of different voltages in the traditional electrophoresis running buffer were statistically analyzed and compared (n = 6). Traditional electrophoresis running buffer resulted in a loss of protein signals as the voltage increased. (**F**) Signal intensities of commercial blocking reagents were statistically analyzed and compared (n = 4). The standard experimental procedure was compatible with commercially blocking reagent, and the protein signal intensity showed a linear relationship with the sample loading mass within a certain scope. (**G**) Modified electrophoresis running buffer tested under different voltage conditions. The experimental procedure was the same as the standard experimental procedure except for the voltage of the modified electrophoresis running buffer.

Considering the role of each component in gel preparation, the compounding scheme can be simplified. We used 10% separating gel (1.5 M Tris (pH 8.8), 1.0 mm) and 5% stacking gel ((1.0 M Tris (pH 6.8), 1.0 mm) as an example. The reagents required for gel preparation were deionized water, 30% Acr-Bis, Tris, 10% SDS, TEMED, and 10% AP. TEMED catalyzes the generation of free radicals from AP, which initiates the crosslinks between bisacrylamide and acrylamide, forming a three-dimensional network. Hence, the isolation of free radicals enables the stable preservation of the mixed solution. Our results show that the first five reagents can be mixed into a system and stored at 4 °C in the dark for a month or more. In this way, the entire compounding steps are simplified to only adding the mixed system and AP. Similarly, dye indicators, which are usually added to form a pre-stained gel for easy sample loading, can also be added to the mixed system without reaction [13]. In order to make the gel agglomeration faster, AP or TEMED could be appropriately adjusted to catalyze the reaction. We compared the traditional step-by-step-added reagent gel, the pre-mixed reagent gel, and the pre-mixed reagent gel at 4 °C overnight. Our results demonstrate no substantial differences in the signal-to-noise ratio among the three gels (Figure 1A,B).

### 2.2. Modification of the Formula of the Running Buffer to Accelerate Electrophoresis

We tested the traditional electrophoresis running buffer (Tris 19.2 mM, glycine 19.2 mM, SDS 3.5 mM, and pH 8.3) at a high voltage. Regrettably, various problems occurred with the voltage increase. One of the most notable drawbacks is that it lost protein signals and could not significantly shorten the electrophoresis time (Figure 1C,E). In order to accelerate the electrophoresis, we modified the formula of the running buffer (Tris 38.1 mM, glycine 266.7 mM, HEPES 21.0 mM, SDS 3.5 mM, and pH 8.3). The modified running buffer could complete electrophoresis within 35 min under 200 V at room temperature (Figure 1D,F) (Table 1). Subsequently, we tested the modified electrophoresis buffer under different voltages (Table 1). Surprisingly, when the voltage was set at 300 V, it still worked normally (Figure 1G), but the ensuing rising heat required an ice-water bath to dissipate it. The 1× electrophoresis buffer was prone to flocculation after storage at room temperature. Certainly, the 10× electrophoresis buffer does not deteriorate easily, but it is difficult to dissolve due to the large amount of electrolytes. Generally, we prepared a 5× electrophoresis buffer for stockpiling.

### 2.3. Replacement of Methanol with Ethanol in the Electrotransfer Buffer to Reduce Toxicity

**Figure 2:**
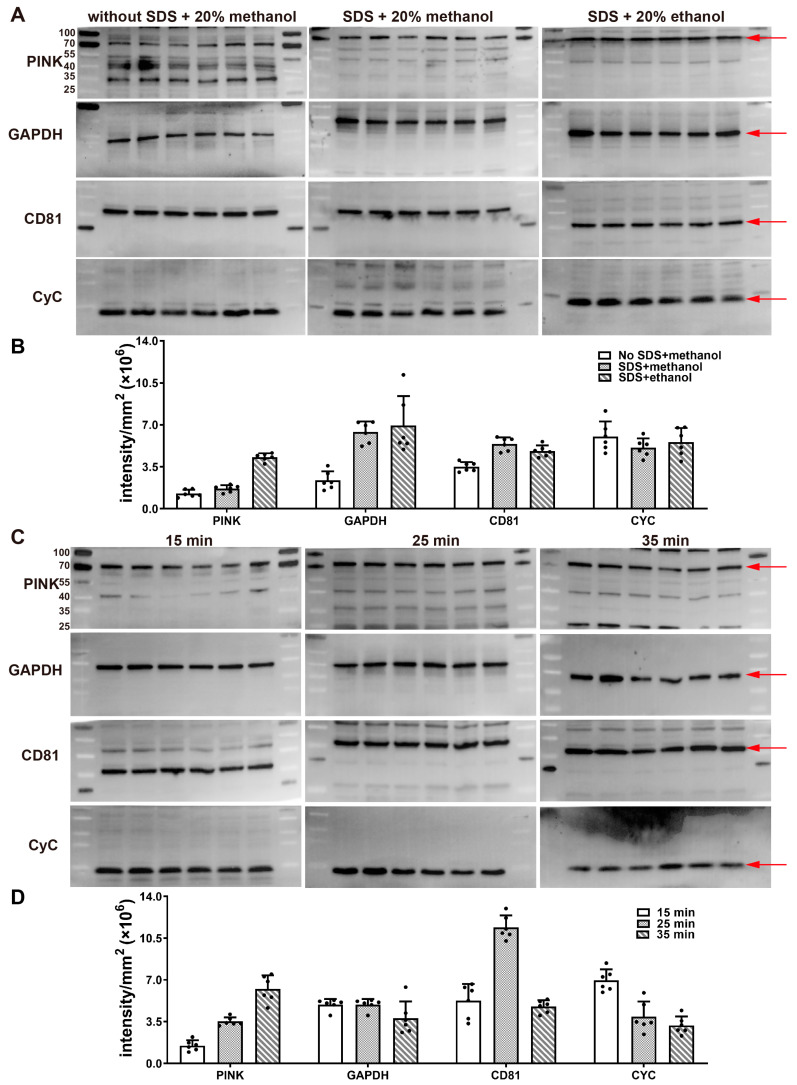
Effect of electrotransfer buffer formula and different electrotransfer times on different molecular weight proteins. (**A**) Replacing methanol with ethanol in the electrotransfer buffer achieved the same efficiency. The experimental procedure was the same as the standard experimental procedure except for the electrotransfer buffer. (**B**) Signal intensities of different electrotransfer buffers were statistically analyzed and compared (n = 6). (**C**) The effect of different electrotransfer times on the signal intensities of proteins with different molecular weights. The experimental procedure was the same as the standard experimental procedure except for the electrotransfer time. (**D**) Signal intensities of different electrotransfer times were statistically analyzed and compared (n = 6).

We replaced the methanol with ethanol in the semi-dry electrotransfer buffer (Tris 36.9 mM, glycine 39.1 mM, SDS 1.3 mM, and 20% ethanol). This upgraded formula decreased methanol vapor. Among the tested proteins, the signal-to-noise ratios of GAPDH, CD81, and CyC were not statistically different between the two groups. However, the signal-to-noise ratio of PINK was the strongest in the ethanol group (Figure 2A,B). In addition, we also tested the effect of SDS on electrotransfer efficiency. Our results imply that the addition of SDS enhanced the signal intensities of GAPDH and CD81 (Figure 2A,B).

### 2.4. Proteins of Different Molecular Weights Have the Most Suitable Electrotransfer Time

For proteins with a sufficiently high abundance, the appropriate extension of the electrotransfer time cannot have a significant effect on the signal intensity. As shown in Figure 2C,D, there was no significant statistical difference in the signal intensity of GAPDH at 15 min, 25 min, and 35 min. However, the signal intensity increased with the extension of electrotransfer time for PINK (70 kDa). In contrast, the signal intensity of CyC (15 kDa) decreased with the extension of the electrotransfer time. When the electrotransfer time was extended to 35 min, the protein marker of CyC could not be visualized (Figure 2C). These results show that proteins with low abundance have relatively suitable electrotransfer times. We extensively tested proteins with 15–130 kDa. Based on our experimental experience, we recommend the setting of the following parameters: 10–25 kDa, 25 V, 15 min; 25–55 kDa, 25 V, 20 min; 55–70 kDa, 25 V, 25 min; and 70–130 kDa, 25 V, 30–35 min.

### 2.5. The 0.45 μm NC Membrane Intercepts Protein Marker Dyes Better Than the 0.45 μm PVDF Membrane

**Figure 3:**
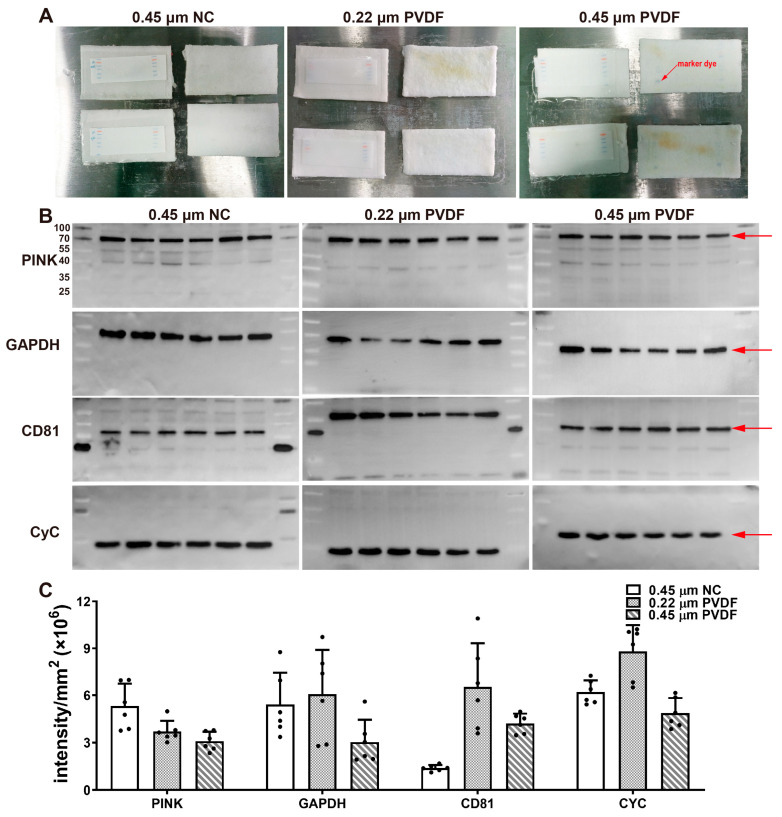
Effects of different membrane materials and pores on protein signal intensities. (**A**) The 0.45 μm PVDF cannot effectively intercept protein marker dye. (**B**) Different membrane materials and pores have different retention capacities for different molecular weight proteins. The experimental procedure was the same as the standard experimental procedure except for the membranes. (**C**) Signal intensities of different materials of membranes or pores were statistically analyzed and compared (n = 6).

We conducted a comparative study on a 0.22 μm PVDF membrane, 0.45 μm PVDF membrane, and 0.45 μm NC membrane. Our results show that the 0.45 μm PVDF could not effectively intercept protein marker dye (Figure 3A), and small-molecular-weight proteins were easily over-transferred when large-molecular-weight proteins were transferred into membranes. The advantage is that the background signal is relatively low. The 0.45 μm NC membrane could intercept protein marker dye (Figure 3A), but it had the same over-transferability issue for small-molecular-weight proteins as the 0.45 μm PVDF membrane. The 0.22 μm PVDF membrane not only retained the pre-stained protein marker but also retained proteins of small and medium molecular weights (Figure 3A–C). Through the quantitative analysis of the signal intensity, our results show that the 0.22 μm PVDF membrane had a significantly stronger interception ability for small-molecular-weight proteins (CyC, CD81) than the 0.45 μm PVDF membrane and 0.45 μm NC membrane (Figure 3B,C).

### 2.6. Cutting the Gel Also Avoids Band Counterfeiting

**Figure 4:**
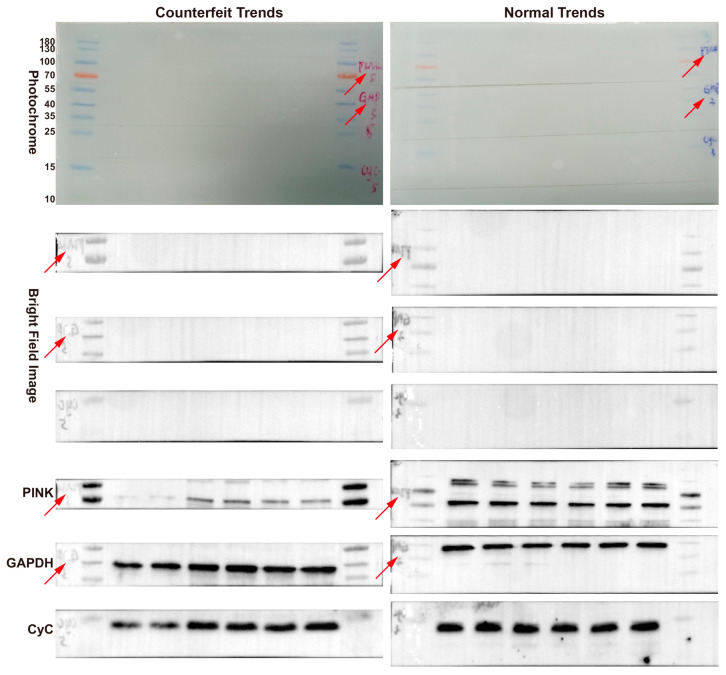
Cutting the gels avoids band counterfeiting.

The immunoblotting band is the hardest-hit area for paper fraud. Every year, myriads of papers are questioned on PubPeer because of protein bands. Gradually, some journals have begun to advocate for uncut gels. Uncut gels undoubtedly increase the workload of researchers and consume reagents and samples. The purpose of an uncut gel is to confirm the target proteins at the corresponding molecular weight and verify the specificity of the primary antibody. With our modified scheme, a cut gel achieved a similar effect. First, we used an oil-based pen to mark the target protein on the membrane after electrotransfer and cut the membrane into bands. Then, the bands were spliced into a whole membrane and an image was acquired. Finally, a brightfield image was preserved during development. As such, photographs plus individual handwriting confirmed the consistency of the bands. As shown in Figure 4, in the bands with falsified trends, the reference protein followed the trend of the target protein. The incompatibility of the reference protein proves that the bands are falsified.

It was confirmed via special handwriting that the three bands originated from the same membrane. By manipulating the total protein mass, we faked the low, high, and medium trends of protein bands, but the reference protein also showed the same trend, proving that there were traces of forgery. Red arrows indicate individual handwriting. The experimental procedure was the same as the standard experimental procedure.

### 2.7. Protein-Free Rapid Blocking Buffer Outperforms Skim Milk

**Figure 5:**
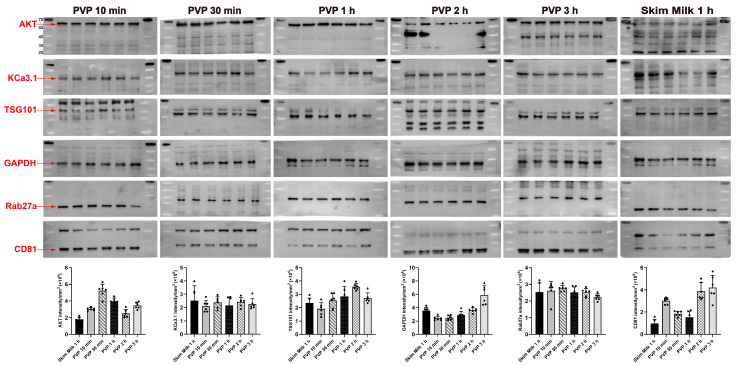
Comparison of the blocking efficiency of PVP-40 and skim milk.

Blocking buffer prevents primary antibody non-specific binding and improves the signal-to-noise ratio. For some high-quality antibodies, blocking may not be performed. We compared the blocking efficiency of polyvinylpyrrolidone (PVP-40) 1% + Tween 20 0.05% with 5% skim milk + Tween 20 0.05%. The signal intensities of AKT, Rab27a, and CD81 were significantly higher in PVP-40 blocking for 10 min than skim milk blocking for 1 h, and the signal intensities of TSG101, KCa3.1, and GAPDH were also not inferior to those of the latter (Figure 5). In addition, commercial blocking buffer did not show overwhelming advantages compared with PVP-40 (Figure 1D). However, aqueous buffers of PVP-40 are prone to spoilage and can only be stored for a week at room temperature. Adding 0.05–1% non-toxic ProClin (Beyotime, Shanghai, China) can greatly extend the shelf life.

The efficiency of PVP-40 blocking for 10 min was comparable to that of skim milk for 1 h. The experimental procedure was the same as the standard experimental procedure except for the blocking reagents (n = 6).

## 3. Discussion

Despite the importance of immunoblotting in biochemical research, the reproducibility of results is poor due to tedious operation and too many uncontrollable variables. To this end, we integrated the existing technologies and solutions to try to produce a guideline that enables beginners to quickly master this skill. Meanwhile, we tried to reduce the experimental cost as much as possible by improving the existing equipment and provided solutions to the current problems.

The concentration, solubility, and abundance of the target protein play a decisive role in the results of immunoblotting. It is necessary to quantify the protein concentration before proceeding to the next step. When the sample contains numerous cells, it is easy to make the sample viscous, which causes the tip of the pipette to be blocked and increases the sample loading error. The main reason for this stickiness is the release of nuclear DNA. Studies have shown that sonication can generate cleavage stress to break down the DNA structure, which dramatically improves the lysis and dissociates the target protein [14,15]. Of course, repeated grinding, pipetting, DNase, or dilution with RIPA and loading buffer (4:1) can also reduce viscosity.

Throughout, reagents are mixed one by one and poured into the gel-making glass plate. Mixing is time-consuming, labor-intensive, and easily confused. We optimized the gel preparation scheme by mixing the reagents into a system, which means that the preparation of the gel is shortened to 10 min. Increasing AP or TEMED by 50–100% significantly accelerates the gel solidification. However, excessive AP or TEMED causes gel burning and distortion of the bands during electrophoresis. The polymerization is fast under alkaline conditions, but the gel is hard and brittle when the alkalinity is too strong. AP or TEMED should be reduced when a high pH is needed.

Traditional electrophoresis requires at least 90 min, which is too time-consuming for high-throughput experiments. Indeed, capillary electrophoresis has revolutionized this field due to its fast speed and high throughput [16,17]. However, it requires the purchase of expensive equipment and custom consumables. This will undoubtedly result in a reluctance to invest in stretched laboratories. Dumut’s laboratory developed a new electrophoresis buffer (100 mM Tris, 100 mM Tricine, and 100 mM HEPES) that could complete electrophoresis in 35 min. Additionally, the resolution of small-molecular-weight proteins could be improved [18]. However, Tricine is not cheap. Our self-developed electrophoresis buffer can significantly shorten the electrophoresis time and effectively separate proteins of different molecular weights. Because of the enhanced ionic strength, the heat increases significantly with the voltage, so the heat dissipation is particularly important. In addition, it is vital to maintain the pH of the electrophoresis buffer. Our results show that the difference in pH between the internal and external buffers after electrophoresis was about 0.3, showing a good buffering performance. Of course, we only tested the buffering capacity of the HEPES, and other “good” buffer systems should theoretically produce excellent results.

Compared with the traditional wet electrotransfer, the efficiency of the semi-dry system is unparalleled. Villanueva’s laboratory has successfully used isopropanol in Towbin’s transfer buffer [19]. Ghanshyam et al. tried to reduce the concentration of methanol. Their results suggested that methanol in electrotransfer buffer had little to no effect on large protein signals. However, a lower concentration of methanol (10%) was sufficient to produce a maximal signal for proteins with small or medium molecular weights [20]. Our research confirmed that ethanol is also reasonable for proteins with small or medium molecular weights. Proteins were efficiently transferred to both NC and PVDF membranes using an ethanol-based electrotransfer buffer. In addition, adding SDS increased the electrotransfer efficiency [21].

Garic et al. developed an electrotransfer buffer (48 mM Tris, 20 mM HEPES, 1.3 mM NaHSO_3_, 1.0 mM EDTA, and 1.3 mM N, N-dimethylformamide) that could be used to complete the electrotransfer in 12 min. NaHSO_3_ compensates for the lack of SDS by acting as a reducing agent, enhances the solubility of large proteins, and acts as a scavenger for free radicals produced by HEPES and HEPPS/EPPS. Furthermore, EDTA indirectly stabilizes piperazine-ring-containing buffers and chelates metal ions. N, N-dimethylformamide acts as a chaotropic agent [22]. Subsequently, Grogery and colleagues filed a patent (US9989493B2) for a rapid electrotransfer buffer (336 mM Tris, 260 mM Glycine, 140 mM Tricine, and 2.5 mM EDTA) that could transfer 10-300 kDa proteins in 5–10 min. This rapid electrotransfer buffer, which is 10 times the ionic strength of traditional transfer buffer, generates considerable heat. Therefore, adding 20% ethanol is effective in dissipating heat.

Interestingly, the pre-stained protein marker could not truly reflect whether the protein was transferred to the 0.45 μm PVDF membrane or not. In the semi-dry system, dye can be transferred to the 0.45 μm PVDF membrane and the lower filter paper simultaneously within 5 min, but almost no protein is transferred to 0.45 μm PVDF membrane. In fact, pre-stained protein markers are mixtures of purified proteins and dyes, which may separate when exposed to an electric field. Hence, whether proteins are transferred to membranes is time-dependent.

Hitherto, academia has still been unable to reconcile the transfer problems of proteins with different molecular weights. Small-molecular-weight proteins move faster than large-molecular-weight proteins, which leads to an asynchronous transfer. The traditional practice transfers proteins of large and small molecular weight separately. In-depth discussions of the reason for this are still taking place. The 0.45 μm PVDF membrane may have a weak retention of fast-moving small-molecular-weight proteins, which can easily penetrate the membrane. Karey et al. reported that using 0.5% glutaraldehyde for the detection of low-molecular-weight acidic and basic isoelectric point proteins increased sensitivity by 1.5-12-fold in immunoblotting [23]. Jing and colleagues proposed that organic solvents and heating substantially avoided the loss of protein signals [24]. Taken together, their methods could lower the detection threshold by enhancing protein binding to membranes. However, the signal enhancement of this fixation method can only be limited to the detection of proteins with a certain molecular weight span. Thus, we propose the following solutions: 1. Reduce the voltage and prolong the electrotransfer time. When the electric field force and the resistance of the PVDF membrane (mechanical resistance and electrostatic force) reach a balance, the protein will not move directionally. 2. Use multiple 0.45 μm PVDF membranes to intercept the transferred protein. When the protein transferred the first 0.45 μm PVDF membrane, the second membrane intercepted the first transferred small-molecular-weight protein within a certain period of time. 3. Reducing the pore size of the PVDF membrane means increasing the resistance. If a 0.22 μm PVDF membrane is used, an extended blocking time may solve the problem of a deep background. However, these solutions can only improve the ability to retain proteins with different molecular weights to a certain extent. Increasing membrane thickness or the stacking of membranes with different pore sizes may provide some new ideas.

The problem that the reference protein and the target protein are not visualized on the same membrane is an inherent defect of immunoblotting. Without the reference protein, these trends can be manipulated at will. Of course, the most effective way is to incubate the same membrane after the primary antibody stripping. However, protein- or antibody-binding properties vary, so stripping conditions need to be explored, which undoubtedly consumes time and effort. This problem can be partly solved using the photography we proposed above. In addition, whole-cell proteins that can be used as reference proteins include GAPDH (37 kDa), Actin (42 kDa), α-Tubulin (50 kDa), β-Tubulin (55 kDa), and HSP90 (90 kDa) [25,26]. When target proteins of different molecular weight needs are cut on the same membrane, the reference protein can be cut in other uncut regions to ensure that each membrane has a reference protein.

Blocking is an effective way to improve the signal-to-noise ratio. Skim milk needs to be prepared immediately because the solution deteriorates rapidly and disguises some primary antibodies. Other substances, such as bovine serum albumin, fish gelatin, and Tween-20, do not make the blocking time shorter [27]. Previous studies found that soymilk is an inexpensive alternative to the commercially available rapid blocking reagent [28]. However, it also possesses the drawback of not being suitable for long-term preservation. PVP-40, which has the properties of nontoxicity and biocompatibility, was reported as a blocking reagent as early as 30 years ago [29]. Cui reported that PVP has the advantage of no or very-low autofluorescence in any of the detection channels. However, it is less effective at blocking non-specific bands [30]. Through a series of time-gradient comparisons, we found that PVP-40 had a good blocking effect even over a short time period. The commercial blocking reagents may not offer a cost-effective advantage, as is shown in Figure 1D.

## 4. Conclusions

We substantially modified the key steps of immunoblotting. First, the simplified gel preparation scheme dramatically increases speed and reduces the chance of failure. Second, the modified electrophoresis buffer formula drastically reduces the time taken while rivalling the traditional formula. Third, replacing methanol with ethanol in the electrotransfer buffer significantly reduces the exposure of laboratory operators to hazardous gases. Finally, PVP-40 achieves similar results while compensating for the shortcomings of skim milk. Compared with the traditional protocol, our standard experimental process improved efficiency by almost four-fold. Our modifications show that even low-quality antibodies can be visualized normally. If the primary antibody is incubated with the membrane at room temperature, the experiment can be completed within one day.

## 5. Materials and Methods

### 5.1. Reagents

Radio Immunoprecipitation Assay Lysis Buffer (RIPA, Servicebio, Wuhan, China), cocktail 100× (100 M PMSF (Beyotime, Shanghai, China), 1 mg/mL leupeptin (Beyotime, Shanghai, China), 1 mg/mL astatin (Beyotime, Shanghai, China)), loading buffer 5× ((1 M Tris 1.25 mL, sodium dodecyl sulfate (SDS) 0.5 g, bromophenol blue 25 mg, 100% glycerol 2.5 mL, β-mercaptoethanol 250 µL) add deionized water to 10 mL), 30% Acr-Bis (acrylamide: bisacrylamide = 29:1), ammonium persulfate (AP, Servicebio, Wuhan, China), tetramethylethylenediamine (TEMED, Servicebio, Wuhan, China), glycine (Biosharp, Beijing, China), Tris (Biosharp, Beijing, China), and 2-[4-(2-hydroxyethyl)piperazin-1-yl] ethanesulfonic acid (HEPES, Biofroxx, Einhausen, Germany). Primary antibodies: Cytochrome C (CyC, 12 KDa, Genetex, Irvine, CA, USA, 1:1000), CD81 (exosome membrane marker, 26 KDa, Abmart, Shanghai, China, 1:1000), Rab27a (exosome secretion-related protein, 28 kDa, Servicebio, Wuhan, China, 1:1000), GAPDH (Glyceraldehyde-3-Phosphate Dehydrogenase, 36 KDa, Servicebio, Wuhan, China, 1:1000), TSG101 (Tumor Susceptibility Gene 101 Protein, 46 kDa, Servicebio, Wuhan, China, 1:1000), KCa3.1 (Potassium Calcium-Activated Channel Subfamily N Member 4, 48 kDa, Proteintech, Wuhan, China, 1:1000), AKT (AKT Serine/Threonine Kinase 1, 60 kDa, Servicebio, Wuhan, China, 1:1000), and PINK (PTEN-Induced Putative Kinase Protein, 63 kDa, Genetex, Irvine, CA, USA, 1:1000). Secondary horseradish peroxidase-conjugated antibody (Proteintech, Wuhan, China, 1:3000), pre-stained protein marker (ThermoFisher, Boston, MA, USA, 26616), 0.22 µm and 0.45 µm polyvinylidene fluoride membrane (PVDF, Millipore, Burlington, MA, USA), 0.45 µm nitrocellulose membrane (NC, Millipore, Burlington, MA, USA), PBS (Biosharp, Beijing, China), Tween-20 (Servicebio, Wuhan, China), and ECL Chemiluminescence Kit (Servicebio, Wuhan, China).

### 5.2. Tissue Lysates

This study was approved by the Animal Studies Subcommittee of our Institutional Review Board. We extracted proteins from 30 mg of C57BL/6J mouse myocardial tissue, adding 800 μL RIPA and 100× cocktail. Then, the tissue was ground twice (JingXin, shanghai, China; 10 Hz, 45 s) and lysed on ice for 10 min. The tissue suspension was centrifuged at 12,000 g for 20 min and the sediment was discarded. The protein concentration was determined by a BCA Protein Assay Kit (Aspen, Wuhan, China), according to the manufacturer’s instructions. The sample was added to 5× loading buffer and boiled at 100 °C for 10 min.

### 5.3. Standard Experimental Procedure

A total of 30 µg per well of total protein was separated by quick gel (Mini Gel). Electrophoresis was performed with the modified electrophoresis running buffer at 200 V for 35 min (Mini-PROTEAN Tetra Cell Systems, Bio-Rad, Los Angeles, CA, USA). Then, proteins were transferred to a 0.45 µm PVDF membrane with ethanol-based electrotransfer buffer with SDS for 25 min (Trans-Blot SD Semi-Dry Electrophoretic Transfer Cell, Bio-Rad, Los Angeles, CA, USA) and blocked with PVP-40 for 10 min. Subsequently, the membrane was incubated with the primary antibody overnight at 4 °C. The membrane was then rinsed three times in PBST. Membranes were incubated with the corresponding secondary antibodies at room temperature for 1 h, rinsed three times with PBST, and then developed (ChemiDoc XRS System, Bio-Rad, Los Angeles, CA, USA).

### 5.4. Analysis of Signal Intensities

The signal intensity of the protein bands was measured and compared using Image Lab (Bio-Rad, Hercules, CA, USA) and GraphPad Prism 8.0 (GraphPad Software, Boston, MA, USA). The density volume within each protein band was measured as intensity/mm^2^. All values are means ± S.E.

## Figures and Tables

**Table 1:** Changes in electrophoresis time and pH of the modified formula at different voltages.

Voltage	Time (RT/LT min)	RT pH (Internal Buffer/External Buffer)	LT pH (Internal Buffer/External Buffer)
150 V	45/55	8.52/8.31	8.47/8.31
200 V	35/40	8.59/8.30	8.48/8.33
250 V	23/29	8.53/8.39	8.59/8.31
300 V	22/27	8.64/8.32	8.57/8.29

Abbreviations: RT, room temperature; LT, low temperature. Note: The electrophoresis time and pH depend on the room temperature. pH was measured by using Sartorius PB-10 (n = 3).

## Data Availability

Not applicable.
